# A Rectal Sunflower Seed Bezoar Causing Fecal Impaction in a Healthy Young Woman

**DOI:** 10.7759/cureus.42317

**Published:** 2023-07-23

**Authors:** Yucel Aydin, Lasha Gogokhia, Syed Mustajab Ahmed, Salim I Turan, Bethel Shiferaw

**Affiliations:** 1 Medicine, Saint Mary's Hospital, Waterbury, USA; 2 Internal Medicine: Infectious Disease, Saint Mary's Hospital, Waterbury, USA

**Keywords:** weight loss, diarrhea, fecal impaction, sunflower seed, bezoars

## Abstract

Bezoars, characterized by undigested or partially digested foreign bodies in the gastrointestinal (GI) tract, are a rare condition associated with significant complications. We present a case of a 31-year-old woman who sought medical attention due to weight loss, diarrhea, and anorectal pain. Upon investigation, she was diagnosed with fecal impaction caused by a rectal sunflower seed bezoar. The patient had a history of regular sunflower seed consumption, suggesting a potential association with the development of the bezoar. Fecal impaction resulting from the bezoar led to chronic constipation, contributing to the patient's weight loss and anorectal discomfort. Imaging studies, including abdominal X-rays or computed tomography (CT) scans, played a crucial role in confirming the diagnosis by identifying the presence of the bezoar within the rectum. Management involved a multidisciplinary approach, with gastroenterologists and colorectal surgeons. A flexible endoscope was utilized to visualize and remove the sunflower seed bezoar under direct vision, providing immediate relief from the symptoms. This case emphasizes the importance of considering bezoars as a potential cause of GI symptoms, even in young and otherwise healthy individuals. Although rectal bezoars are relatively rare, they can lead to significant morbidity. Therefore, it is essential to include them in the differential diagnosis of patients presenting with fecal impaction, anorectal pain, and associated symptoms. Prompt diagnosis, appropriate imaging, and early intervention are crucial for the successful management and prevention of potential complications and the improvement of patient outcomes.

## Introduction

A bezoar is a solid mass formed by the accumulation of indigestible or poorly digestible substances within the gastrointestinal (GI) tract, commonly found in the stomach and small intestine [1]. Different types of bezoars are classified based on the materials from which they originate, such as trichobezoars (hair), phytobezoars (plant fiber), pharmacobezoars (ingested drugs), and lactobezoars (milk protein). Among them, phytobezoars composed of indigestible fruit and vegetable matter, including fiber, peels, and seeds, are the most prevalent [2].

Bezoars typically develop in individuals with certain risk factors, such as mental retardation, psychiatric disorders like trichotillomania (compulsive hair-pulling) or trichotillophagia (compulsive hair-eating), inadequate chewing, abnormal gastric emptying (as seen in diabetes mellitus or mixed connective tissue disorders), and altered GI anatomy resulting from procedures like gastric bypass or partial gastrectomy accompanied by vagotomy [3].

In many cases, bezoars are asymptomatic and are incidentally discovered during endoscopic procedures performed for unrelated reasons. However, as they migrate downward through the GI tract with peristalsis, bezoars can become more symptomatic. The symptoms associated with bezoars can vary depending on their location and the presence of complications, such as gastric outlet obstruction, GI bleeding due to ulceration, ileus, perforation, and intussusception [4]. Gastric bezoars often lead to symptoms such as postprandial fullness, abdominal pain, nausea, vomiting, anorexia, and weight loss. On the other hand, small intestinal bezoars commonly cause bowel obstruction. Rectal bezoars can result in constipation, rectal ulcers, and rectal bleeding secondary to fecal impaction.

In mild to moderate cases of bezoars, medical treatment with enzymatic agents like cellulase or dark soda is typically attempted as the initial approach. However, more severe or refractory cases may require interventions such as manual disimpaction, endoscopic removal, or surgery [5]. In this report, we present an exceedingly rare case involving a rectal phytobezoar composed of sunflower seeds, which led to fecal impaction and subsequent symptoms including overflow diarrhea, weight loss, rectal ulceration, and anorectal pain.

## Case presentation

**Figure 1:**
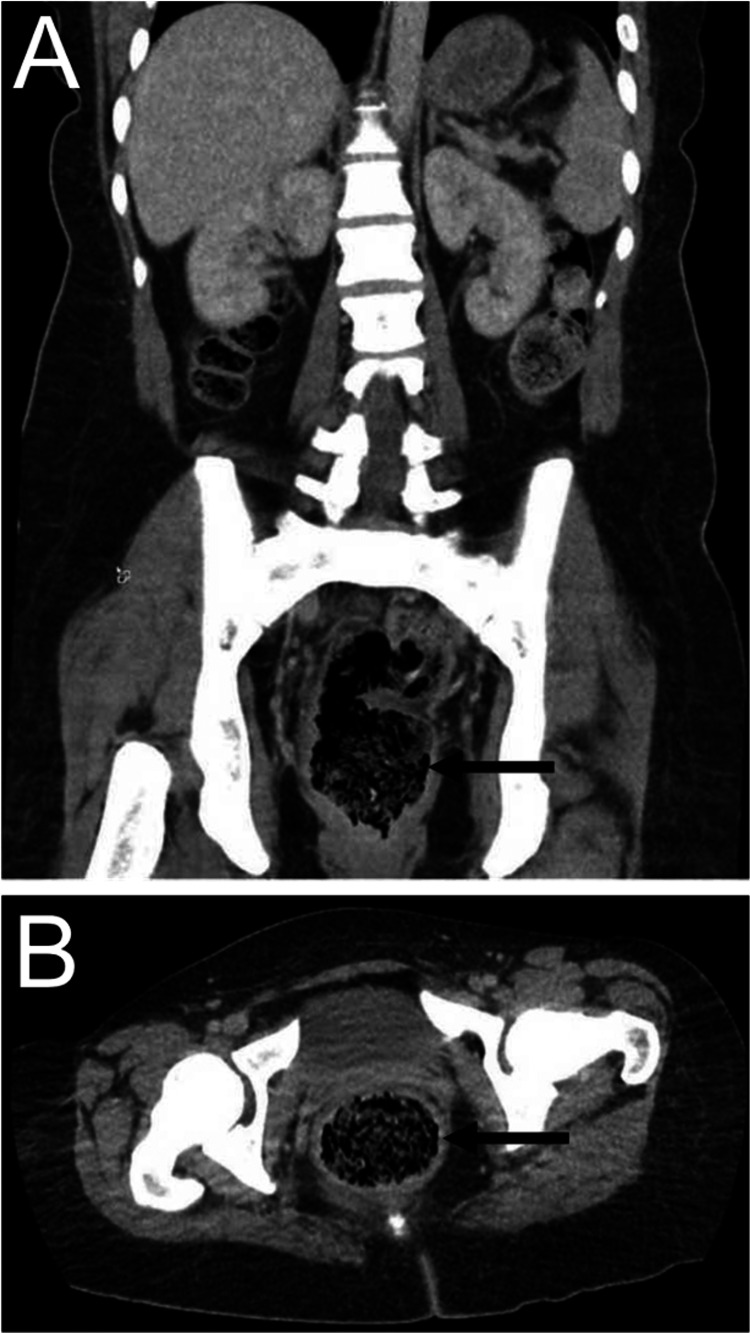
Coronal (A) and axial (B) sections of the CT abdomen and pelvis. Arrows indicate fecal impaction in the rectum.

A 31-year-old woman with no significant past medical history presented to the clinic with severe watery diarrhea, along with a weight loss of 20 pounds over a span of four weeks. She complained of anorectal pain and exhibited a perianal rash. The onset of diarrhea coincided with the initiation of stool softeners four weeks prior, which she had started taking for constipation. The patient denied any previous episodes of diarrhea or constipation, and there were no reports of fever, nausea, vomiting, recent travel, sick contacts, or anal intercourse. On physical examination, the patient appeared unremarkable, except for a heart rate of 128 beats per minute. The perianal area exhibited an erythematous, superficial rash, along with dried sloughed-off external hemorrhoids. Due to severe anorectal tenderness, the digital rectal examination was not performed. Laboratory data and stool studies yielded unremarkable results, except for iron deficiency anemia. To further investigate the condition, a computed tomography (CT) scan of the abdomen and pelvis was conducted, revealing fecal impaction and the presence of hyperdense material within the rectosigmoid colon, as observed in Figure 1A, 1B.

**Figure 2:**
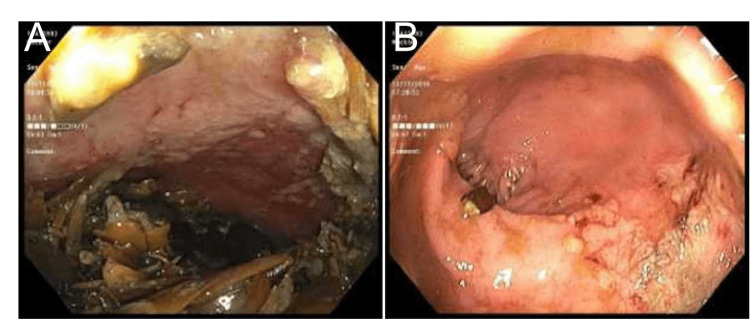
The rectum with a sunflower seed bezoar leading to a mass effect during colonoscopy (A) and an endoscopic image of rectal ulcer secondary to the sunflower seed bezoar (B).

Upon obtaining a detailed history from the patient, it was revealed that she had been consuming a large quantity of sunflower seeds. Consequently, a colonoscopy was performed under general anesthesia. The colonoscopy revealed the presence of a large phytobezoar consisting of undigested seeds, accompanied by multiple superficial ulcerations and edematous, friable mucosa in the surrounding area, indicative of fecal impaction (Figure 2A, 2B).

**Figure 3:**
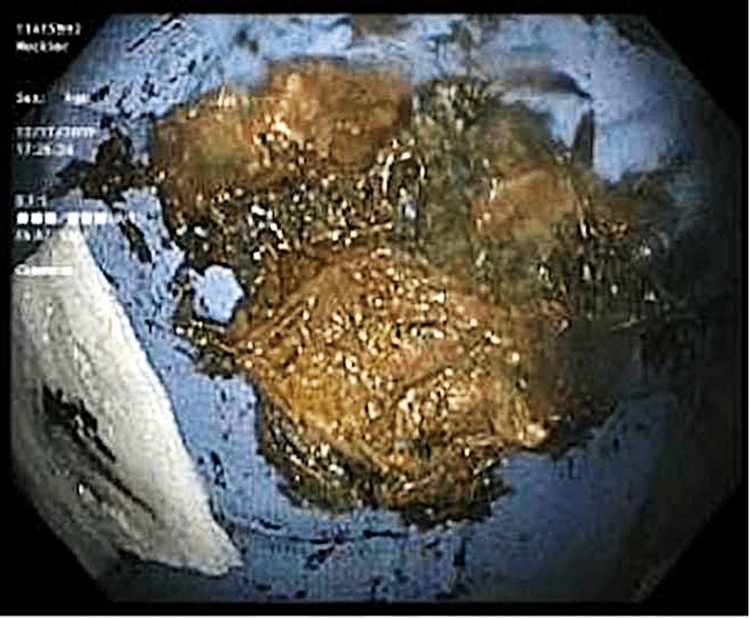
The sunflower seed bezoar causing fecal impaction. It was removed manually under general anesthesia.

The sigmoid colon and the rest of the colon appeared normal. During the procedure, successful manual disimpaction was achieved, leading to rapid alleviation of pain and discomfort (Figure 3).

Subsequently, the patient was discharged with instructions to follow a high-fiber diet and refrain from consuming sunflower seeds.

The presented case highlights the association between excessive sunflower seed consumption and the development of a rectal phytobezoar, leading to fecal impaction. In patients with a history of constipation or signs of obstruction, the possibility of overflow diarrhea should be considered as part of the differential diagnosis, particularly in the presence of increased abdominal distension. The patient experienced significant symptoms, including severe watery diarrhea, substantial weight loss, anorectal pain, and the presence of a perianal rash. Prompt identification and intervention, in the form of manual disimpaction during colonoscopy, resulted in the rapid resolution of symptoms. The patient was advised to modify her dietary habits by incorporating a high-fiber diet and avoiding further sunflower seed consumption. 

## Discussion

Bezoars are relatively rare GI tract disorders that often remain silent and asymptomatic, particularly in individuals with predisposing factors. The stomach is the most common location for bezoar formation, followed by the small intestine. In this case, we present a rare occurrence of a sunflower seed bezoar in the rectum, leading to fecal impaction, rectal ulcers, and anorectal pain in a young woman without any identifiable precipitating factors. It is noteworthy that our patient experienced severe diarrhea and significant weight loss following the use of a stool softener for constipation.

Bezoars are relatively uncommon, with an average of 2.5 cases per year reported in high-volume patient centers [6]. While sunflower seed bezoars are more commonly observed in children who consume seeds with shells, only a few cases of rectal seed bezoars have been reported in adults. Rectal seed bezoars appear to arise in patients without known predisposing risk factors, such as connective tissue disorders, dysmotility, or strictures, similar to our patient [7]. The pathophysiological mechanism underlying the formation of seed bezoars in the GI tract differs from other types of bezoars. Seed bezoars, a unique subclass of phytobezoars, typically pass through the pylorus and ileocecal valve and accumulate in the colon and rectum, where they further dehydrate and harden, leading to impaction, ulceration, and anal pain. Among seeds, watermelon seeds are reported as the most common cause of bezoars, followed by sunflower seeds, according to a recent review on bezoars [8].

CT imaging plays a crucial role in both identifying impacted bezoars and excluding other potential etiologies, such as rectal cancer or obstruction. Following successful bezoar evacuation, a complete colonoscopy is recommended to assess for possible strictures and colon ulcers. While small rectal bezoars may pass spontaneously or with the aid of enemas, symptomatic large rectal bezoars require manual rectal disimpaction under general anesthesia. Surgical intervention is indicated when conservative management, including manual or endoscopic disimpaction, fails or when complications such as perforation are present [9]. Additionally, secondary prevention is crucial to prevent future recurrences by identifying and addressing underlying risk factors. In the case of our patient, we recommended discontinuing sunflower seed consumption and adopting a high-fiber diet to promote optimal bowel function and prevent further bezoar formation.

## Conclusions

Bezoars are a rare condition that may be unfamiliar to many physicians; however, maintaining a high index of suspicion in patients with predisposing factors and GI symptoms is crucial for diagnosis. A rectal sunflower seed bezoar is an exceedingly rare entity that presents with constipation, anorectal pain, and rectal bleeding. Accurate diagnosis relies on meticulous history-taking and digital rectal examination, complemented by CT abdomen and colonoscopy in selected cases. Long-term management following bezoar detection necessitates addressing underlying risk factors to minimize the likelihood of recurrence.
